# Disitamab Vedotin in HER2-Positive and HER2-Low Breast Cancer: A Multicenter Retrospective Analysis

**DOI:** 10.32604/or.2025.065029

**Published:** 2025-08-28

**Authors:** Xizhou Zhang, Zetao Zhang, Jianguang Lin, Jiarong Yi, Xuxiazi Zou, Jikun Feng, Guangsheng Huang, Bingfeng Chen, Junxi Long, Fengjia Wu, Feng Ye, Haoming Wu

**Affiliations:** 1 The Breast Center, Cancer Hospital of Shantou University Medical College, Guangdong Provincial Key Laboratory of Breast Cancer Diagnosis and Treatment, Shantou, 515031, China; 2 Department of Oncology, The Second Affiliated Hospital of Fujian Medical University, Quanzhou, 362000, China; 3 State Key Laboratory of Oncology in South China, Collaborative Innovation Center for Cancer Medicine, Sun Yat-sen University Cancer Center, Guangzhou, 510060, China

**Keywords:** Disitamab vedotin, antibody-drug conjugates, real-world study, breast cancer, HER2-low, HER2-RC48, ADC

## Abstract

**Background:**

Breast cancer remains a leading cause of morbidity and mortality among women worldwide, with significant geographic disparities in its impact. While human epidermal growth factor receptor 2 (HER2)-targeted therapies, such as trastuzumab, have improved outcomes for HER2-positive breast cancer, challenges like therapy resistance persist, highlighting the need for novel treatments. Recent developments in antibody-drug conjugates (ADCs), particularly disitamab vedotin (RC48), show promising efficacy in targeting both HER2-positive and HER2-low expression tumors, warranting further investigation through real-world studies to assess its broader clinical applicability.

**Method:**

This retrospective, multicenter observational study evaluated the real-world efficacy and safety of RC48 in patients with HER2-positive or HER2-low breast cancer across three medical centers in China. Patient demographic characteristics, treatment patterns, sequential use of ADCs, and treatment-related adverse events were recorded and analyzed.

**Result:**

The median progression-free survival (mPFS) for the overall population (*n* = 96) was 4.31 months, with HER2-positive patients demonstrating significantly longer mPFS (5.26 months) compared to HER2-low patients (3.45 months; *p* = 0.044), while subgroup analyses revealed no significant differences in mPFS based on estrogen receptor (ER), progesterone receptor (PR), or hormone receptor (HR) status. Safety data indicated that adverse events were consistent with prior reports, with no new safety concerns identified during the study period.

**Conclusion:**

This real-world study demonstrates the efficacy of RC48 in both HER2-positive and HER2-low breast cancer. Notably, combination therapy significantly improved outcomes in HER2-low patients.

## 1 Introduction

Breast cancer, a leading global malignancy, represents a formidable public health challenge for women worldwide. As reported in Global Cancer Statistics 2022, this disease constituted 11.6% of newly diagnosed cancers and 6.9% of global cancer-related mortality in 2022 [1]. Although advancements in early detection and precision therapeutics have reduced age-standardized mortality rates, profound geographical inequities endure, with regions of low socio-demographic indices bearing disproportionately elevated mortality rates [2].

The heterogeneity of breast cancer subtypes complicates treatment outcomes, particularly for human epidermal growth factor receptor 2 (HER2)-positive breast cancers, which account for 14% of cases and are associated with a poor prognosis [3]. While HER2-targeted therapies like trastuzumab have transformed treatment paradigms, persistent challenges such as innate or acquired drug resistance highlight the urgent demand for innovative approaches [4–8].

Antibody-drug conjugates (ADCs) have recently emerged as groundbreaking agents that synergize monoclonal antibody specificity with potent cytotoxic payloads, optimizing tumor-selective cytotoxicity while sparing healthy tissue [9,10]. Disitamab vedotin (RC48), a novel HER2-targeted ADC, demonstrates therapeutic versatility beyond conventional HER2-positive classifications (immunohistochemistry (IHC) 3+ or IHC 2+/fluorescence *in situ* hybridization (FISH)-positive), extending efficacy to HER2-low malignancies (IHC 1+/2+ without amplification) [11–14]. Preliminary trials reveal RC48’s promising activity in HER2-low advanced breast cancer, achieving objective response rates (ORR) exceeding 30% [13].

Despite these encouraging findings, much of the evidence supporting RC48’s use stems from controlled clinical trial settings, which may not fully capture real-world complexities. Recent real-world studies have begun to address this gap. Wang et al. examined RC48 in 89 metastatic breast cancer patients with different HER2 expression levels, demonstrating superior progression-free survival (PFS) in HER2-positive vs. HER2-low patients [6.6 vs. 4.1 months] [15]. Similarly, Qu et al. conducted a five-center study of 154 patients, reporting a median progression-free survival (mPFS) of 5.06 months with higher objective response rate in HER2-positive patients, while also suggesting benefits of combination therapy over monotherapy [16].

Our current multicenter retrospective study seeks to provide further critical insights. We extend beyond previous work by incorporating detailed subgroup analyses designed to elucidate factors influencing RC48 outcomes. A specific contribution of our research is the investigation into how prior therapeutic strategies, notably including prior treatment with programmed death-1 (PD-1) inhibitors, affect patient response to RC48-ADC. This approach aims to generate more nuanced real world evidence to better inform the strategic use of RC48-ADC in complex clinical scenarios. By addressing these knowledge gaps, this analysis seeks to provide a more comprehensive understanding of RC48’s role in the evolving landscape of breast cancer management, particularly in clinical scenarios that extend beyond the scope of controlled trials and earlier real-world investigations.

## 2 Method

### 2.1 Study Design and Patients

This investigation was a retrospective, multicenter observational study designed to evaluate the real-world efficacy and safety of RC48 in patients diagnosed with HER2-positive or HER2-low breast cancer. Data were collected from three participating medical centers in China: The Cancer Hospital of Shantou University Medical College, Sun Yat-sen University Cancer Center, and the Second Affiliated Hospital of Fujian Medical University. The study cohort comprised individuals who received at least one cycle of RC48 across three participating medical centers in China between 01 April 2021, and 31 December 2024. To mitigate selection bias, consecutive enrollment was used, including all patients who met the inclusion criteria during the study period. Follow-up data were systematically collected until 31 December 2024. The reporting of this study follows the Strengthening the Reporting of Observational Studies in Epidemiology (STROBE) guidelines.

### 2.2 Eligibility Criteria

The inclusion criteria were as follows: (1) age ranging from 18 to 85 years; (2) histological confirmation of HER2-positive or HER2-low breast cancer; (3) an Eastern Cooperative Oncology Group (ECOG) performance status score within the range of 0–2; and (4) receipt of at least one cycle of RC48 treatment. Patients with incomplete medical documentation or insufficient follow-up data were excluded from this analytical cohort.

### 2.3 Ethical Considerations

Ethical approval was duly obtained from the Institutional Review Boards (IRBs) by Cancer Hospital of Shantou University Medical College (No. 2025020). The study was conducted in strict adherence to the principles outlined in the Declaration of Helsinki. Due to the retrospective nature of the study, the requirement for informed consent was waived.

### 2.4 Data Acquisition and Definitions

Data were meticulously extracted from electronic health records, pathology reports, and treatment logs by two rigorously trained investigators. Any discrepancies encountered were adjudicated by a third, senior reviewer. Baseline demographic, clinical, and treatment-related data were retrieved from electronic medical records at each participating center.

### 2.5 Efficacy Endpoints

Efficacy outcomes were evaluated based on the PFS and overall survival (OS). PFS was calculated from the date of RC48 therapy initiation to the date of disease progression or death from any cause. OS was defined as the interval from the commencement of RC48 treatment to death from any cause.

### 2.6 Safety Assessment

Safety data were compiled to assess treatment-related adverse events (TRAE), which were graded according to the Common Terminology Criteria for Adverse Events (CTCAE) version 5.0. TRAE were categorized by severity and organ system involvement.

### 2.7 Statistical Analyses

All statistical analyses were performed using R software, version 4.4.1. Descriptive statistics were employed to summarize baseline demographic and clinical characteristics. Continuous variables were expressed as means with standard deviations (SD) or medians with interquartile ranges (IQR), and categorical variables were presented as frequencies and percentages. Kaplan-Meier survival analysis was utilized to estimate PFS and OS, with inter-subgroup differences assessed via the log-rank test. To identify factors potentially associated with survival outcomes, univariate Cox proportional hazards regression analysis was performed for each potential predictor. Results were presented as hazard ratios (HR) and their corresponding 95% confidence intervals (CI). TRAE were analyzed descriptively, with incidence rates calculated for all recorded events. Comparisons of TRAE frequencies across patient subgroups were performed using chi-square or Fisher’s exact tests, as deemed appropriate. A two-sided *p*-value of less than 0.05 was considered statistically significant.

## 3 Result

### 3.1 Baseline Characteristics

A cohort of 96 patients diagnosed with HER2-positive or HER2-low breast cancer was included in the baseline analysis. The study population was stratified by HER2 expression status into HER2-low (*n* = 46) and HER2-positive (*n* = 50) subgroups. Baseline demographic and clinical characteristics are comprehensively detailed in Table 1. The overall cohort exhibited a mean age of 52.58 years (SD = 9.89) and a mean body mass index (BMI) of 21.87 (SD = 3.33), with no statistically significant intergroup disparities in age (*p* = 0.248) or BMI (*p* = 0.075). However, menopausal status differed significantly, with all HER2-low subgroups being post-menopausal (100%) compared to 80% of the HER2-positive subgroup (*p* = 0.001). The HER2-positive subgroup exhibited a greater incidence of prior exposure to HER2-targeted therapies (76.1% vs. 12.0%, *p* < 0.001) and tyrosine kinase inhibitors (TKIs) (76.1% vs. 30.0%, *p* < 0.001). Conversely, the HER2-low subgroup more frequently had prior exposure to endocrine therapy (58.0% vs. 19.6%, *p* < 0.001), cyclin-dependent kinases (CDK) inhibitors (90.0% vs. 39.1%, *p* < 0.001), and antiangiogenic therapy (98.0% vs. 78.3%, *p* = 0.003). Bone metastasis incidence was significantly higher in the HER2-low subgroup (62.0% vs. 37.0%, *p* = 0.024), while no notable between-group differences emerged in other metastatic sites, treatment lines (≥3 lines: 76.1% vs. 64.0%, *p* = 0.266), or combination therapy (67.4% vs. 60.0%, *p* = 0.527).

**Table 1:** Baseline characteristics of patients with HER2-positive and HER2-low breast cancer

Characteristics	Total ( *n* = 96)	HER2-positive ( *n* = 50)	HER2-low ( *n* = 46)	*p*-values
Age [mean (SD), years]	52.58 (9.89)	51.46 (10.76)	53.80 (8.81)	0.248
BMI [mean (SD), kg/m^2^]	21.87 (3.33)	22.45 (3.37)	21.24 (3.20)	0.075
Number of treatment lines [median (IQR)]	4 (2, 7)	3 (2, 7)	4 (3, 7)	0.391
Menstrual status [n (%)]				0.001
Pre-menopausal	10 (10.4)	10 (20.0)	0 (0.0)	
Post-menopausal	86 (89.6)	40 (80.0)	46 (100.0)	
Family history of tumors (%)				0.187
Yes	10 (10.4)	3 (6.0)	7 (15.2)	
No	86 (89.6)	47 (94.0)	39 (84.8)	
Comorbidities (%)				0.819
Yes	24 (25.0)	12 (24.0)	12 (26.1)	
No	72 (75.0)	38 (76.0)	34 (73.9)	
Prior treatment (%)				0.243
Yes	93 (96.9)	47 (94.0)	46 (100.0)	
No	3 (3.1)	3 (6.0)	0 (0.0)	
Prior radiotherapy (%)				0.297
Yes	37 (38.5)	22 (44.0)	15 (32.6)	
No	59 (61.5)	28 (56.0)	31 (67.4)	
Prior chemotherapy (%)				1
Yes	91 (94.8)	47 (94.0)	44 (95.7)	
No	5 (5.2)	3 (6.0)	2 (4.3)	
Prior endocrine therapy (%)				<0.001
Yes	58 (60.4)	21 (42.0)	37 (80.4)	
No	38 (39.6)	29 (58.0)	9 (19.6)	
Prior PD-1 inhibitors use (%)				0.024
Yes	11 (11.5)	2 (4.0)	9 (19.6)	
No	85 (88.5)	48 (96.0)	37 (80.4)	
Prior CDK inhibitors use (%)				<0.001
Yes	33 (34.4)	5 (10.0)	28 (60.9)	
No	63 (65.6)	45 (90.0)	18 (39.1)	
Prior HER2-targeted therapy (%)				<0.001
Yes	55 (57.3)	44 (88.0)	11 (23.9)	
No	41 (42.7)	6 (12.0)	35 (76.1)	
Prior TKIs use (%)				<0.001
Yes	46 (47.9)	35 (70.0)	11 (23.9)	
No	50 (52.1)	15 (30.0)	35 (76.1)	
Prior anti-angiogenic therapy (%)				0.003
Yes	11 (11.5)	1 (2.0)	10 (21.7)	
No	85 (88.5)	49 (98.0)	36 (78.3)	
Prior ADCs use (%)				1
Yes	19 (19.8)	10 (20.0)	9 (19.6)	
No	77 (80.2)	40 (80.0)	37 (80.4)	
Prior mTOR inhibitors use (%)				0.049
Yes	4 (4.2)	0 (0.0)	4 (8.7)	
No	92 (95.8)	50 (100.0)	42 (91.3)	
ER status (%)				0.004
Positive	65 (67.7)	27 (54.0)	38 (82.6)	
Negative	31 (32.3)	23 (46.0)	8 (17.4)	
PR status (%)				0.024
Positive	55 (57.3)	23 (46.0)	32 (69.6)	
Negative	41 (42.7)	27 (54.0)	14 (30.4)	
HR status (%)				0.012
Positive	69 (71.9)	30 (60.0)	39 (84.8)	
Negative	27 (28.1)	20 (40.0)	7 (15.2)	
Ki67 index(%)				0.101
High	90 (93.8)	49 (98.0)	41 (89.1)	
Low	6 (6.2)	1 (2.0)	5 (10.9)	
Number of metastatic sites (%)				0.822
<3	70 (72.9)	37 (74.0)	33 (71.7)	
≥3	26 (27.1)	13 (26.0)	13 (28.3)	
Bone metastasis (%)				0.024
Yes	48 (50.0)	19 (38.0)	29 (63.0)	
No	48 (50.0)	31 (62.0)	17 (37.0)	
Liver metastasis (%)				0.102
Yes	47 (49.0)	20 (40.0)	27 (58.7)	
No	49 (51.0)	30 (60.0)	19 (41.3)	
Brain metastasis (%)				0.486
Yes	25 (26.0)	15 (30.0)	10 (21.7)	
No	71 (74.0)	35 (70.0)	36 (78.3)	
Lung metastasis (%)				0.673
Yes	35 (36.5)	17 (34.0)	18 (39.1)	
No	61 (63.5)	33 (66.0)	28 (60.9)	
Treatment lines (%)				0.266
<3	29 (30.2)	18 (36.0)	11 (23.9)	
≥3	67 (69.8)	32 (64.0)	35 (76.1)	
Combined therapy (%)				
Yes	61 (63.5)	30 (60.0)	31 (67.4)	0.527
No	35 (36.5)	20 (40.0)	15 (32.6)	

Note: SD, standard deviations; IQR, interquartile ranges; HER2, human epidermal growth factor receptor-2; BMI, body mass index; PD-1 inhibitors, programmed death-1 inhibitors; CDK Inhibitors, cyclin-dependent kinases inhibitors; TKIs, tyrosine kinase inhibitors; ADCs, antibody-drug conjugates; mTOR, mammalian target of rapamycin; ER, estrogen receptor; PR, progesterone receptor; HR, hormone receptor.

### 3.2 Overall Population

**Figure 1:**
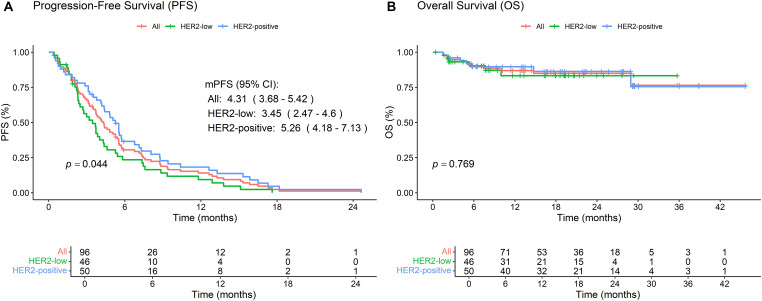
Kaplan-Meier survival analyses of patients treated with RC48 in breast cancer. **(A)** Progression-free survival (PFS) curves stratified by HER2 status, showing outcomes for both HER2-positive and HER2-low subgroups. **(B)** Overall survival (OS) curves stratified by HER2 status, comparing survival outcomes between HER2-positive and HER2-low patients. The log-rank test was used to compare survival outcomes between subgroups

For the entire cohort, the mPFS was 4.31 months (95% CI: 3.68–5.42) (Fig. 1A). Stratification by HER2 status revealed a significant difference in mPFS, with the HER2-low subgroup exhibiting a shorter mPFS of 3.45 months (95% CI: 2.47–4.6) compared to 5.26 months (95% CI: 4.18–7.13) in the HER2-positive subgroup (*p* = 0.044). Median overall survival (mOS) remained non-estimable due to the limited number of events (*n* = 13) and the relatively short follow-up period up to 31 December 2024 (Fig. 1B). Given the current immaturity of OS data, caution is warranted in interpreting the survival outcomes, and extended follow-up is planned to better characterize the long-term survival benefit of RC48.

**Figure 2:**
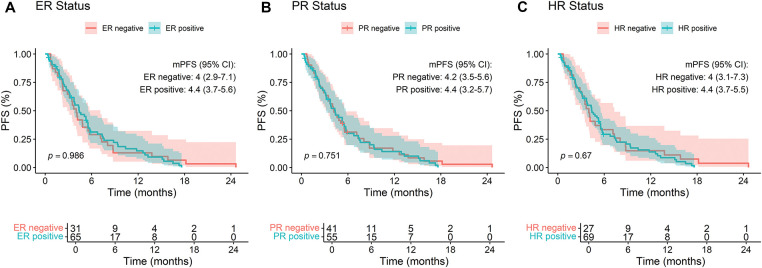

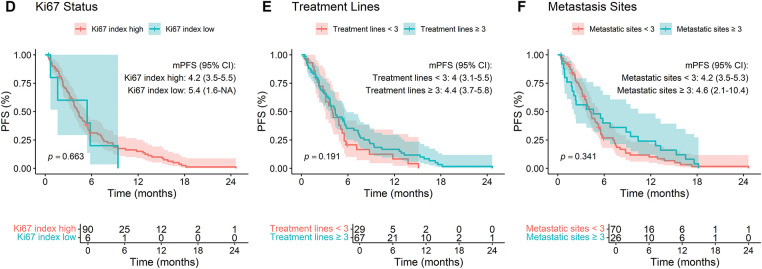
Kaplan-Meier analysis of progression-free survival (PFS) stratified by **(A)** ER status, **(B)** PR status, **(C)** HR status, **(D)** Ki67 index, **(E)** lines of treatment, and **(F)** number of metastatic sites in breast cancer patients treated with RC48

Subgroup analyses indicated no statistically significant differences in PFS based on estrogen receptor (ER) status (negative = 4.0 vs. positive = 4.4, *p* = 0.986), progesterone receptor (PR) status (negative = 4.2 vs. positive = 4.4, *p* = 0.751), or hormone receptor (HR) status (negative = 4.0 vs. positive = 4.4, *p* = 0.67) (Fig. 2A–C). Similarly, Ki67 index (high = 4.2 vs. low = 5.4, *p* = 0.663), number of prior treatment lines (<3 lines = 4.0 vs. ≥3 lines = 4.4, *p* = 0.191), and metastatic sites (<3 sites = 4.2 vs. ≥3 sites = 4.6, *p* = 0.341) did not significantly influence PFS (Fig. 2D–F).

### 3.3 HER2-Positive Subgroup

**Figure 3:**
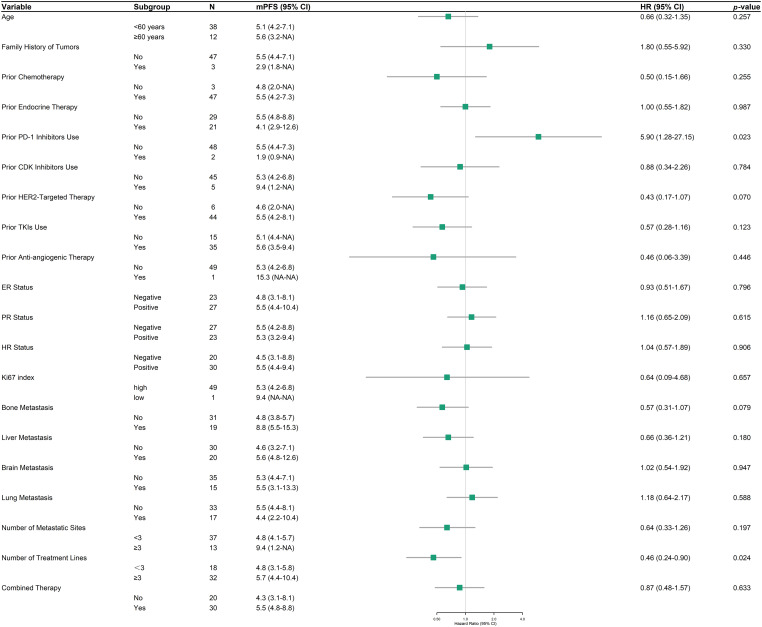
Forest plot of progression-free survival (PFS) in HER2-positive patients across subgroups

Within the HER2-positive cohort (*n* = 50), a focused subgroup analysis of PFS was conducted to discern factors associated with RC48 efficacy (Fig. 3). Prior programmed death-1 (PD-1) inhibitors use (HR = 5.90, 95% CI: 1.28–27.15, *p* = 0.023), and the number of treatment lines (HR = 0.46, 95% CI: 0.24–0.90, *p* = 0.024) demonstrated statistically significant associations with PFS. In contrast, other evaluated variables, including age, family tumor history, and metastatic site distribution, did not reach statistical significance. However, emerging trends suggested potential benefits in patients with bone metastases (HR = 0.57, 95% CI: 0.31–1.07, *p* = 0.079) and prior HER2-targeted drug exposure (HR = 0.43, 95% CI: 0.17–1.07, *p* = 0.070). HR status, Ki67 index, and combined therapy showed no discernible impact on PFS.

### 3.4 HER2-Low Subgroup

**Figure 4:**
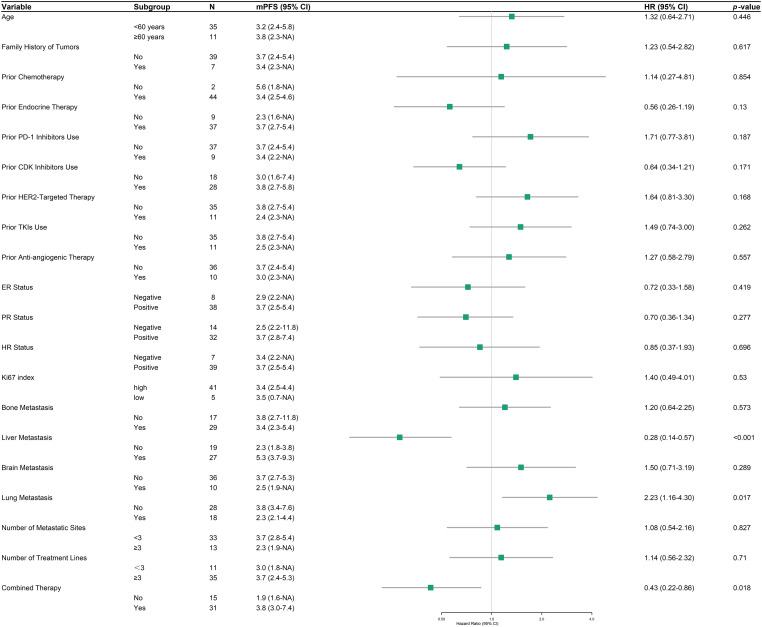
Forest plot of progression-free survival (PFS) in HER2-low patients across subgroups

In the HER2-low subgroup analysis, PFS and HR were assessed across diverse clinical and demographic variables to identify potential predictors of treatment response to RC48 (Fig. 4). Liver metastasis was associated with a substantially prolonged PFS (*n* = 27, mPFS 5.3 months, 95% CI: 3.7–9.3) compared to cases without liver involvement (*n* = 19, mPFS 2.3 months, 95% CI: 1.8–3.8; HR = 0.28, 95% CI: 0.14–0.57, *p* < 0.001). Conversely, lung metastasis correlated with diminished outcomes (2.3 vs. 3.8 months; HR = 2.23, 95% CI: 1.16–4.30, *p* = 0.017). Combination therapy significantly enhanced PFS (3.8 months, 95% CI: 3.0–7.4) compared to monotherapy (1.9 months, 95% CI: 1.6–NA; HR = 0.43, 95% CI: 0.22–0.86, *p* = 0.018). Other examined factors, including age, family history of tumors, and prior treatment history, did not exhibit statistically significant associations with PFS.

### 3.5 Treatment Patterns

**Figure 5:**
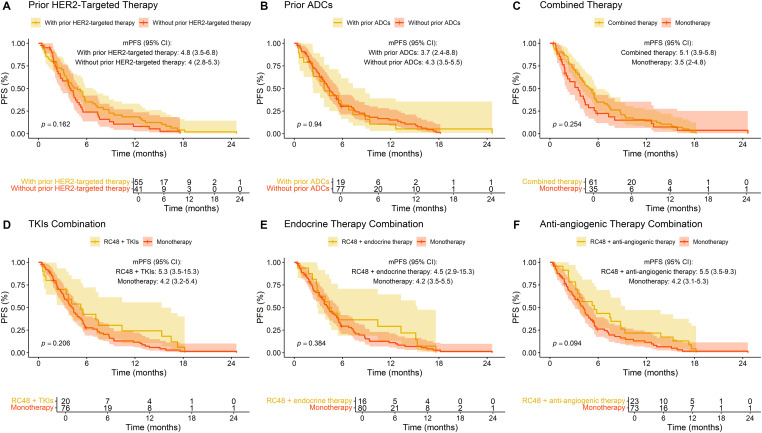
Progression-free survival (PFS) stratified by prior treatment and combination therapies in breast cancer patients treated with RC48. **(A)** Prior HER2-targeted therapy; **(B)** prior ADCs exposure; **(C)** combined therapy; **(D)** TKIs combination; **(E)** endocrine therapy combination; **(F)** anti-angiogenic agents combination

A survival analysis was performed to evaluate the influence of various treatment patterns on PFS (Fig. 5). Patients with prior HER2-targeted therapy demonstrated an mPFS of 4.8 months (95% CI: 3.5–6.8) compared to 4.0 months (95% CI: 2.8–5.3) in those without (*p* = 0.16). Similarly, prior ADCs use did not significantly affect PFS, with values of 3.7 months (95% CI: 2.4–8.8) and 4.3 months (95% CI: 3.5–5.5) for patients with and without prior ADCs, respectively (*p* = 0.94). RC48 combined therapy trended toward improved PFS (5.1 vs. 3.5 months; *p* = 0.25). Notably, combining RC48 with anti-angiogenic drugs yielded the most extended PFS (5.5 vs. 4.2 months; *p* = 0.094), approaching borderline significance. No significant survival differences were observed when combining RC48 with TKIs or endocrine therapy (*p* = 0.21 and *p* = 0.38, respectively).

### 3.6 Sequential ADCs Utilization

**Figure 6:**
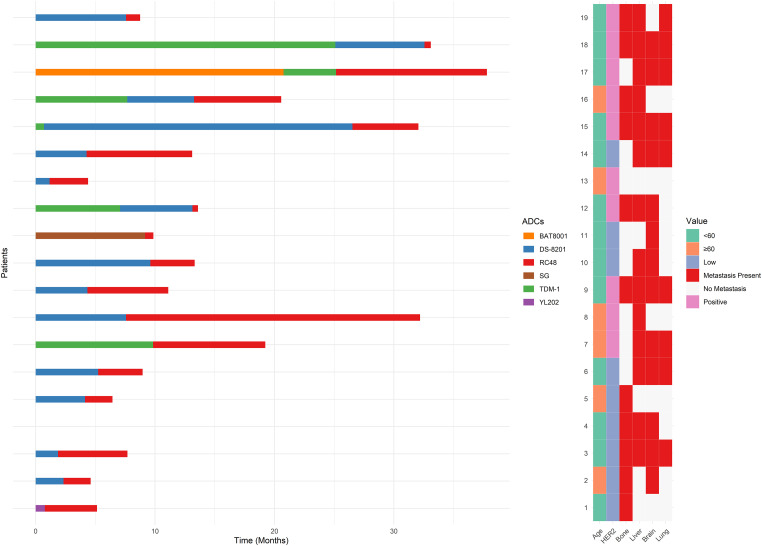
Swimmer plot of patients receiving RC48 after treatment with other antibody-drug conjugates (ADCs)

Fig. 6 illustrates the outcomes of RC48 treatment following prior use of other ADCs. Notably, patients who had previously received two or more ADCs exhibited a mPFS of 5.52 months, compared to 3.72 months for those with only one prior ADCs (*p* = 0.304). Among specific prior ADCs, patients treated with RC48 after trastuzumab deruxtecan (DS-8201) had a mPFS of 3.72 months, while those previously treated with trastuzumab emtansine (TDM-1) exhibited a longer mPFS of 6.41 months. Individual cases included prior use of sacituzumab govitecan (SG), BAT8001, and YL202, with mPFS values of 0.69, 12.63, and 4.37 months, respectively (Data not explicitly shown in Fig. 6).

### 3.7 Safety

The overall incidence of TRAE among all patients was 28.1% (27/96), suggesting that nearly one-third of the cohort experienced at least one adverse event (Table 2). The majority of TRAE were mild to moderate (grade 1–2), with severe (grade ≥3) TRAE occurring in 5.2% (5/96) of cases. These included one instance of alanine aminotransferase (ALT) increased and four cases of bone marrow suppression. The most frequently reported adverse reactions were platelet count decreased (7.3%), pain (6.3%), and white blood cell (WBC) decreased (5.2%). Bone marrow suppression, encompassing WBC decreased (5/96), neutrophil count decreased (2/96), lymphocyte count decreased (4/96), platelet count decreased (7/96), and anemia (3/96), emerged as the most prevalent category of adverse events.

**Table 2:** Treatment-related adverse events (TRAE) in all patients

TRAE	Total (*n* = 96)	Grade 3/4 (*n* = 96)	Her2-Low (*n* = 46)	Her2-Positive (*n* = 50)	*p*-Values	Age < 60 (*n* = 73)	Age ≥ 60 (*n* = 23)	Monotherapy (*n* = 35)	Combined Therapy (*n* = 61)
Total (%)	27 (28.1)	5 (5.2)	12 (26.1)	15 (30.0)	0.670	19 (26.0)	8 (34.8)	10 (28.6)	17 (27.9)
Neurotoxicity (%)	4 (4.2)		1 (2.2)	3 (6.0)	0.670	2 (2.7)	2 (8.7)	3 (8.6)	1 (1.6)
Gastrointestinal reactions (%)	3 (3.1)		1 (2.2)	2 (4.0)	1.000		3 (13.0)		3 (4.9)
Rash (%)	3 (3.1)		2 (4.3)	1 (2.0)	0.941	1 (1.4)	2 (8.7)	1 (2.9)	2 (3.3)
Gingival bleeding (%)	2 (2.1)		1 (2.2)	1 (2.0)	1.000	1 (1.4)	1 (4.3)	1 (2.9)	1 (1.6)
Alopecia (%)	2 (2.1)			2 (4.0)	0.512	1 (1.4)	1 (4.3)	1 (2.9)	1 (1.6)
Pain (%)	6 (6.3)		1 (2.2)	5 (10.0)	0.246	3 (4.1)	3 (13.0)	2 (5.7)	4 (6.6)
Fatigue (%)	2 (2.1)		1 (2.2)	1 (2.0)	1.000		2 (8.7)		2 (3.3)
Weight loss (%)	1 (1.0)			1 (2.0)	1.000	1 (1.4)			1 (1.6)
Blurred vision (%)	1 (1.0)		1 (2.2)		0.479		1 (4.3)		1 (1.6)
WBC decreased (%)	5 (5.2)	1 (1.0)	2 (4.3)	3 (6.0)	1.000	2 (2.7)	3 (13.0)	2 (5.7)	3 (4.9)
Platelet count decreased (%)	7 (7.3)	1 (1.0)	3 (6.5)	4 (8.0)	1.000	3 (4.1)	4 (17.4)	2 (5.7)	5 (8.2)
Anemia (%)	3 (3.1)	1 (1.0)	1 (2.2)	2 (4.0)	1.000	2 (2.7)	1 (4.3)		3 (4.9)
Lymphocyte count decreased (%)	4 (4.2)		3 (6.5)	1 (2.0)	0.551	3 (4.1)	1 (4.3)		4 (6.6)
Neutrophil count decreased (%)	2 (2.1)	1 (1.0)	1 (2.2)	1 (2.0)	1.000	1 (1.4)	1 (4.3)		2 (3.3)
AST increased (%)	3 (3.1)		1 (2.2)	2 (4.0)	1.000	1 (1.4)	2 (8.7)	1 (2.9)	2 (3.3)
ALT increased (%)	2 (2.1)	1 (1.0)	1 (2.2)	1 (2.0)	1.000	1 (1.4)	1 (4.3)	1 (2.9)	1 (1.6)
GGT increased (%)	2 (2.1)		1 (2.2)	1 (2.0)	1.000	1 (1.4)	1 (4.3)		2 (3.3)

Note: TRAE, treatment-related adverse events; WBC, white blood cell; AST, aspartate aminotransferase; ALT, alanine aminotransferase; GGT, gamma-glutamyl transferase: *p*-values represent the comparison of TRAE between the HER2-low and HER2-positive subgroups.

A comparative analysis of TRAE incidence between HER2-positive and HER2-low breast cancer patients revealed no statistically significant differences. The HER2-positive group exhibited a TRAE incidence of 30.0% (15/50), while the HER2-low group showed a slightly lower rate of 26.1% (12/46). HER2-positive patients were more susceptible to pain, neurotoxicity, and alopecia, whereas the HER2-low group experienced fewer adverse reactions overall, with blurred vision uniquely observed in one case within this subgroup.

Further stratification by age demonstrated that patients aged ≥60 years had a TRAE incidence of 34.8% (8/23), compared to 26.0% (19/73) in those aged <60 years, with no significant statistical disparity. However, older patients (≥60 years) exhibited a higher propensity for gastrointestinal reactions and fatigue.

Therapeutic regimen comparison showed equivalent TRAE rates between monotherapy (28.6%, 10/35) and combined therapy recipients (27.9%, 17/61). Nevertheless, combined therapy was associated with heightened occurrences of bone marrow suppression, gastrointestinal reactions, and pain relative to monotherapy.

## 4 Discussion

The emergence of ADCs has catalyzed a transformative evolution in breast cancer management, particularly for HER2-positive and HER2-low expression malignancies [17,18]. RC48, a novel HER2-targeting ADC, has exhibited remarkable clinical efficacy across trials, demonstrating substantial antitumor activity even in the challenging population of HER2-low advanced breast cancer [13,19,20]. Nevertheless, validating these trial findings in real-world clinical practice remains imperative to assess their generalizability across heterogeneous patient demographics. This retrospective, multicenter observational study aimed to evaluate the clinical efficacy and safety of RC48 in patients with HER2-positive or HER2-low breast cancer.

The mPFS in our cohort was 4.31 months, with HER2-positive patients experiencing significantly longer mPFS compared to HER2-low patients (5.26 vs. 3.45 months, *p* = 0.044). These results are consistent with prior studies that have shown superior efficacy of HER2-targeted therapies in HER2-positive breast cancer, likely attributable to higher HER2 expression levels, which enhance ADCs binding and internalization [13,16]. However, the mPFS observed in this study (5.26 months) was marginally shorter than previously reported (6.6 months or 5.93 months), potentially due to variations in patient characteristics, treatment regimens, or follow-up durations. The efficacy observed in HER2-low patients suggests that RC48 may exert antitumor effects through mechanisms beyond HER2-dependent internalization, such as bystander killing, a phenomenon previously reported with other ADCs like DS-8201 [21].

Subgroup analyses further revealed that prior PD-1 inhibitors use was associated with significantly shorter PFS in HER2-positive patients (HR = 5.90, *p* = 0.023). Previous research has demonstrated that the combination of RC48 with PD-1 inhibitors yields favorable outcomes in HER2-positive breast cancer [22–24]. Further studies are warranted to investigate the mechanisms by which the prior use of PD-1 inhibitors affects the efficacy of RC48. Conversely, the association between a higher number of prior treatment lines and improved PFS is counterintuitive and may be confounded by unmeasured factors in our analysis (HR = 0.46, *p* = 0.024). It is plausible that patients who tolerated and responded to multiple prior treatment lines were inherently more robust and thus better positioned to benefit from RC48.

In the HER2-low subgroup, liver metastases correlated with prolonged PFS, whereas lung metastases were linked to poorer outcomes. While this observation aligns with a previous real-world study in which HER2-low breast cancer patients with liver metastases exhibited longer PFS, we acknowledge several potential confounding factors that may influence these results [16]. Patients with liver metastases often receive more intensive supportive care, undergo more frequent imaging surveillance, and may have different baseline characteristics that could impact treatment outcomes. These factors warrant careful consideration when interpreting site-specific treatment efficacy, and this finding merits further investigation through controlled studies. Notably, RC48 demonstrated promising efficacy in a preclinical mouse model of HER2-positive breast cancer with lung metastases [25].

Combined therapy has emerged as a pivotal determinant of RC48 efficacy, particularly in patients with low HER2 expression. The integration of RC48 with other therapeutic agents significantly extended mPFS to 3.8 months, compared to 1.9 months with monotherapy (HER2-low: *p* = 0.018). Regarding the combination with anti-angiogenic therapy, a numerical increase in mPFS was observed compared to the control group (5.5 vs. 4.2 months), although this difference did not reach statistical significance (*p* = 0.094). The exploration of this combination is supported by preclinical evidence underscoring the advantages of combining targeted agents with complementary mechanisms of action [26,27]. For instance, Mirvetuximab soravtansine (IMGN853), an FRα-targeting ADCs, exhibited synergy with bevacizumab in platinum-resistant ovarian cancer models, inducing rapid disruption of tumor microvasculature and extensive necrosis [26]. Such vascular normalization or destruction may augment ADCs delivery to tumor cells by reducing interstitial fluid pressure and enhancing intratumoral drug penetration, offering a plausible explanation for the observed trend in our study [28]. However, the combination of RC48 with TKIs or endocrine therapy failed to yield significant benefits, emphasizing the necessity for biomarker-driven strategies to refine combination regimens [29].

In addition to anti-angiogenic therapy, innovative combination regimens have shown promise in further enhancing RC48 efficacy. One such regimen is the PRaG3.0 protocol, which combines RC48-ADC with hypofractionated radiotherapy (HFRT), PD-1/programmed death-ligand 1 (PD-L1) inhibitors, sequential granulocyte-macrophage colony-stimulating factor (GM-CSF), and interleukin-2 (IL-2) [30,31]. This approach is considered a potentially effective salvage therapy for advanced solid tumor patients expressing HER2, demonstrating manageable safety and encouraging antitumor activity (mPFS = 6.3–7.2 months) [32,33]. While this regimen was not extensively evaluated in our cohort, its favorable outcomes highlight the importance of exploring multimodal strategies that integrate immunomodulatory agents and localized therapies to maximize the therapeutic potential of RC48.

Furthermore, we observed a notable trend in the limited subset of patients receiving RC48 in combination with chemotherapy. Among the four patients treated with this combination, the mPFS reached 7.36 months, surpassing the overall median PFS observed in other combination strategies. However, the small sample size precludes definitive conclusions and highlights the need for larger studies to validate whether this represents a true synergistic effect or selection bias toward chemotherapy-sensitive populations.

Our analysis of sequential ADCs use has uncovered some intriguing patterns. Patients who received two or more prior ADCs demonstrated numerically longer PFS (5.52 months) compared to those with only one prior ADCs (3.72 months), although this difference did not reach statistical significance (*p* = 0.304). Among specific ADCs, prior exposure to TDM-1 was associated with superior PFS (6.41 months) relative to DS-8201 (3.72 months). These observations align with emerging data suggesting that the efficacy of sequential ADCs therapy varies according to payload characteristics and mechanisms of action [34–38]. While limited by sample size and statistical significance, these findings provide preliminary real-world evidence suggesting RC48 could be a potential subsequent treatment option following TDM-1 and DS-8201. However, the small sample size for specific prior ADCs, such as SG and BAT8001, precludes definitive conclusions. Given the distinct payload mechanisms of these ADCs, variations in resistance mechanisms may influence the efficacy of sequential treatments [39]. Further research, particularly larger prospective or real-world studies, is warranted to confirm these findings and determine optimal ADC sequencing strategies, including the role of RC48 after agents like T-DM1 and DS-8201.

The safety profile of RC48 in this study was consistent with previous reports, characterized by manageable hematologic and gastrointestinal toxicities [13,40,41]. The incidence of TRAE was lower than in prior clinical trials, further corroborating the drug’s manageable toxicity profile [13,40,41]. Notably, no cardiac toxicity or interstitial pneumonia was observed in our study. This finding is consistent with two previous real-world studies on RC48 that similarly reported an absence of cardiac adverse events [15,16]. In contrast, other HER2-targeted ADCs, including DS-8201 and TDM-1, have demonstrated cardiac toxicity in previous clinical investigations [42,43]. Our analysis did not identify any novel TEAE beyond those previously documented, further validating the established safety profile of RC48. The accumulating evidence suggesting a lower incidence of cardiac toxicity with RC48 indicates that this agent may represent a preferred therapeutic option for patients with pre-existing cardiac conditions or those at increased risk for cardiotoxicity. However, the observed absence of cardiac toxicity in this study may be influenced by patient selection criteria. Specifically, patients with significant cardiac comorbidities were excluded from our study population, which could contribute to the favorable cardiac safety profile reported here. To address this, future studies with broader inclusion criteria, or those specifically designed to evaluate cardiac safety in patients with cardiac risk factors, are warranted to fully confirm the cardiac safety of RC48. Nevertheless, comprehensive safety analyses, including long-term follow-up data, are essential to better define toxicity risks and potential mitigation strategies. While retrospective data provide preliminary insights, the inherent limitations of such studies in TRAE capture emphasize the need for prospective validation.

Several limitations warrant acknowledgment. Firstly, as a retrospective study, it is inherently susceptible to various biases, including selection bias and recall bias. The selection bias arises because the inclusion of patients was based on available records, which might not fully represent the broader population of HER2-positive or HER2-low breast cancer patients, potentially leading to an over- or underestimation of RC48’s efficacy and safety. Recall bias may also occur due to reliance on historical records and patient recall, resulting in possible underreporting or misclassification of adverse events and other clinical outcomes. Although rigorous data validation measures were implemented to mitigate these biases, residual confounding factors cannot be entirely eradicated. Secondly, the modest sample size constrained the statistical power necessary to discern nuanced differences in subgroup analyses, especially concerning rare TRAE or infrequent clinical scenarios. Lastly, the lack of OS maturity further restricts the ability to draw definitive conclusions.

Future investigations should prioritize validating these findings through larger, prospective studies with extended follow-up periods. Comprehensive biomarker analyses, including detailed genomic profiling and assessments of HER2 expression levels, are essential for identifying predictive markers of RC48 response and elucidating the underlying mechanisms of observed disparities among HER2 subgroups and metastatic sites. Furthermore, randomized controlled trials comparing various combination strategies are imperative to establish evidence-based guidelines for optimizing RC48 therapy.

## 5 Conclusion

In conclusion, this real-world study substantiates the clinical efficacy of RC48 in both HER2-positive and HER2-low breast cancer, with differential therapeutic responses correlated to prior treatment exposure and metastatic site distribution. While these findings harmonize with existing evidence, prospective validation is critical to confirm optimal therapeutic sequencing and combination strategies. Future research should aim to further elucidate the biological mechanisms underlying treatment response and resistance, ultimately guiding more personalized treatment approaches for patients with breast cancer.

## Data Availability

The data that support the findings of this study are available from the Corresponding Author, [Haoming Wu], upon reasonable request.

## Abbreviations

HER2: human epidermal growth factor receptor 2
ADCs: antibody-drug conjugates
RC48: disitamab vedotin
IHC: immunohistochemistry
FISH: fluorescence *in situ* hybridization
ORR: objective response rates
STROBE: Strengthening the Reporting of Observational Studies in Epidemiology
ECOG: Eastern Cooperative Oncology Group
IRBs: Institutional Review Boards
PFS: progression-free survival
OS: overall survival
TRAE: treatment-related adverse events
CTCAE: Common Terminology Criteria for Adverse Events
SD: standard deviations
IQR: interquartile ranges
HR: hazard ratios
CI: confidence intervals
BMI: body mass index
TKIs: tyrosine kinase inhibitors
CDK: cyclin-dependent kinases
mPFS: median progression-free survival
mOS: median overall survival
ER: estrogen receptor
PR: progesterone receptor
HR: hormone receptor
PD-1: programmed death-1
mTOR: mammalian target of rapamycin
DS-8201: trastuzumab deruxtecan
TDM-1: trastuzumab emtansine
SG: sacituzumab govitecan
AST: aspartate aminotransferase
ALT: alanine aminotransferase
GGT: gamma-glutamyl transferase
WBC: white blood cell
IMGN853: Mirvetuximab soravtansine
HFRT: hypofractionated radiotherapy
PD-L1: programmed death-ligand 1
GM-CSF: granulocyte-macrophage colony-stimulating factor
IL-2: interleukin-2
